# Effects of Pressure, Surfactant Concentration, and Heat Flux on Pool Boiling Using Expanding Microchanneled Surface for Two-Phase Immersion Cooling

**DOI:** 10.3390/ma17215155

**Published:** 2024-10-23

**Authors:** Yifei Hu, Dengwei Fu, Chaobin Dang, Sihui Hong

**Affiliations:** 1 School of Physics and Astronomy, Sun Yat-sen University, Zhuhai 519082, China; huyf63@mail2.sysu.edu.cn (Y.H.); fudw3@mail2.sysu.edu.cn (D.F.); 2 Graduate School of Engineering, University of Fukui, 3-9-1 Bunkyo, Fukui-shi, 910-8507, Fukui, Japan; dangcb@u-fukui.ac.jp; 3 College of Electric Engineering, Zhejiang University, Hangzhou 310027, China

**Keywords:** two-phase immersion cooling, sub-atmospheric pressure, non-ionic surfactants, temperature oscillation, bubble behaviors, boiling heat transfer

## Abstract

Deionized water is replacing fluorinated liquids as the preferred choice for two-phase immersion cooling in data centers. Yet, insufficient bubble removal capability at low saturated pressure is a key challenge hindering the widespread application. To solve this issue, this study employs non-ionic surfactant (Tween 20) and asymmetric structures (expanding microchannel) to enhance the boiling performances of deionized water under sub-atmospheric pressure. The research examines the effects of pressure (8.8~38.5 kPa), surfactant concentration (0.1~0.5 mL/L), and heat flux density (10~180 W/cm^2^) on the boiling heat transfer characteristics and analyzes the mechanism of unusual temperature oscillations induced by surfactants. It was found that the trade-off between the sub-atmospheric pressure, surface tension coefficient, and reduced static contact angle results in pronounced intermittent boiling on the heated surface. Even with the addition of surfactants, the improvement in heat transfer requires demanding conditions. Boiling enhancement throughout all heat flux conditions was achieved when the surfactant concentration was higher than 0.2 mL/L for the expanding microchanneled surface. The heat transfer coefficient reached 6.89 W·cm^−2^·K^−1^ under 8.8 kPa, which was 45% higher than without the surfactant. Under the same heat flux and sub-atmospheric pressure, as the concentration increased from 0.1 to 0.5 mL/L, the amplitudes of temperature fluctuation of the plane surface and expanding microchanneled surface decreased from 10 K to 2 K and 18 K to 1 K, respectively. The onset of nucleate boiling and wall superheat of the expanding microchanneled surface gradually decreased with the increase in surfactant concentration, where the onset of nucleate boiling decreased by 10.54 K. When the heat flux is 160 W/cm^2^, the wall superheat is reduced by 12.8 K.

## 1. Introduction

### 1.1. Challenges of Two-Phase Immersion Cooling in Data Centers

Data centers consume enormous amounts of energy, making them high-energy-consuming industries. Energy-efficient green data centers typically require a Power Usage Effectiveness (PUE) of less than 1.3, while the PUE of air-cooled or air-conditioned systems is often above 1.7 [1]. Further optimization and improvements bring limited marginal benefits, failing to meet energy consumption requirements. Immersion liquid cooling systems are a thermal management technology in which electronic devices or other heat-generating components are fully immersed in a specially designed liquid coolant with a PUE lower than 1.1, minimizing system overheating, temperature fluctuations, fan failures, noise, dust, and air quality issues [2]. This enhances the reliability and lifecycle of data center systems, making immersion liquid cooling the most promising technology for data center cooling systems [3]. Immersion liquid cooling systems can be divided into single-phase liquid cooling and two-phase liquid cooling, depending on whether the cooling liquid undergoes phase change. Single-phase liquid cooling uses high-boiling-point (>100 °C) cooling liquids such as mineral oil or perfluorinated polyether, with lower cooling efficiency but reliable performance, currently at a high level of commercialization. Two-phase immersion cooling exponentially improves the heat transfer efficiency of the cooling liquid through the boiling and condensation processes. However, due to reliability issues with phase-change systems, actual commercial applications have not yet been realized. However, there are some initial applications, such as Microsoft’s use of 3M liquid coolant in 2021, where the boiling liquid carries away heat generated by computer servers in Microsoft’s data centers [4].

As mentioned before, compared to traditional cooling techniques, two-phase immersion cooling [5,6,7,8] has garnered increasing attention due to its high heat transfer efficiency. However, the development of this technology currently faces two main technical bottlenecks. First is the insufficient bubble removal rate. At low heat flux, the bubble detachment frequency that solely relies on buoyancy is low, resulting in poor boiling heat transfer performances. At high heat flux (>100 W/cm^2^), the vapor generation rate sharply increases, rapidly forming a vapor film covering the heating surface [9,10]. Steam leaves in the form of continuous vapor columns that overlap each other, hindering the wetting of the liquid to the heating components. Mutual interference between the vapor and liquid phases leads to the “dry-out” of micro-liquid layers, resulting in a sharp deterioration of boiling heat transfer [11,12]. The second issue is the mismatch of fluid properties. Electronic fluorinated liquids have good insulation properties but poor heat transfer performance [13], with an evaporation latent heat only 5% to 10% of that of deionized (DI) water. Moreover, some suitable electronic fluorinated liquids have been prohibited worldwide as they are not friendly to the environment. It is exigent and important to explore available alternatives for thermal management utilizing liquid-cooling techniques. DI water, replacing fluorinated liquids as the primary working fluid, is preferred due to its high latent heat, environmental friendliness, and economic benefits. The use of water cooling has gradually become the primary choice for many data centers [14]. Moreover, existing technologies allow for the insulation of electronic devices from DI water [13]. However, the issue of high boiling temperature (>100 °C) using DI water in electronic cooling needs to be addressed. Low saturated pressure conditions greatly reduce the onset temperature of the phase change [15], which enables a reasonable operating temperature for two-phase immersion cooling. However, at low pressure (<101 kPa), the interfacial Gibbs free energy is significantly higher, and the diameter of detached bubbles increases by more than 10 times compared to atmospheric pressure conditions. This exacerbates the difficulty of bubble removal, leading to excessively low boiling heat transfer performance. Meanwhile, intermittent boiling occurs due to increased surface tension and decreased vapor density [16], leading to significant surface temperature fluctuations [17]. Currently, it is still challenging to apply this technology to two-phase immersion liquid-cooling systems. This study considers the use of additives (surfactants) in combination with machined surfaces (expanded channel structures) to enhance heat transfer under low-pressure conditions.

### 1.2. State of the Art on Pool Boiling Enhancement Using Surfactant

Pool boiling refers to the process of heating a liquid within a confined volume to its boiling point, resulting in the formation of bubbles on the heated surface and heat being transferred. As a critical process in immersion, phase-change cooling achieves efficient heat transfer through phenomena such as nucleation, bubble growth, and detachment on the heated surface. Therefore, studying how to enhance the pool boiling effect under different conditions is of great significance for improving the overall efficiency of immersion phase-change cooling. In studies of various pool boiling enhancement techniques, the use of surfactants as additives in water under atmospheric pressure has been proven to be highly effective [18,19,20,21]. The enhancement mechanism primarily involves two aspects [22,23,24,25,26]: firstly, reducing surface tension to facilitate bubble detachment, and secondly, the interaction of hydrophilic and hydrophobic groups at the bubble interface to prevent bubble coalescence. The heat transfer enhancement is closely related to the concentration and type of surfactant. The maximum enhancement is generally achieved when the concentration is close to the critical micelle concentration (CMC) [25], and the types mainly differ among cationic, anionic, and non-ionic surfactants. It is reported that the anionic surfactant enhanced the boiling heat transfer efficiency of the nanofluid, with its heat transfer coefficient surpassing that of the cationic surfactant, which in turn exceeded that of the nonionic surfactant [27]. 

However, there are some inconsistencies in the literature regarding the role of surfactants in enhancement, making the effect less predictable. Yang [28], Lee [29], and Proper et al. [30] pointed out that the addition of surfactants reduces equilibrium surface tension, thereby enhancing boiling heat transfer, but also reduces the static contact angle, inhibiting part of the heat transfer process. Additionally, Yang et al. [31] and Jia et al. [32] observed that under high heat flux conditions, during the boiling of surfactant solutions, a phenomenon of bubble crowding occurs, where bubble clusters expand and contract. Periodic temperature fluctuations and heat transfer deterioration could occur.

Moreover, under low saturated pressure, enhancing boiling heat transfer with surfactants faces significant challenges. Bubble behavior is closely related to heat transfer performance, but as pressure decreases, surface tension increases and vapor density decreases, resulting in larger bubble detachment diameters and a corresponding reduction in bubble detachment frequency [16,33]. Under such conditions, intermittent boiling phenomena may occur. Giraud et al. [34] pointed out that boiling phenomena under sub-atmospheric pressure (0.8 kPa~15 kPa) are influenced by environmental non-uniformity, defining an “intermittent boiling regime”, with wall temperature fluctuations reaching up to 20 K. Yamada et al. [35] reported that under sub-atmospheric pressure, when the heat flux is 6 W/cm^2^, deionized water exhibits continuous boiling when the saturation pressure exceeds 14 kPa. However, as the pressure decreases from 11 kPa to 8.8 kPa, temperature fluctuations intensify, indicating a transition towards intermittent boiling.

In conclusion, under low-pressure conditions, the difficulty of using surfactants as a means of heat transfer enhancement significantly increases, particularly in maintaining stable boiling and preventing heat transfer deterioration, which may lead to unstable temperature oscillations and intermittent boiling. Previous studies on the impact of surfactants on pool boiling heat transfer have primarily focused on atmospheric pressure conditions, with relatively few investigations into the effects of surfactant concentration on pool boiling performance under low-pressure conditions. 

**Figure 1:**
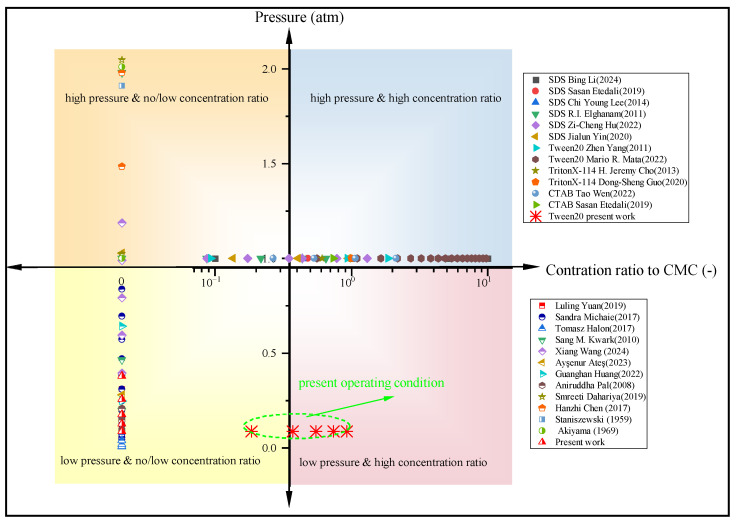
Experimental data under different working conditions.

Table 1 summarizes the comparisons of various studies under different conditions, listing the experimental conditions at different sub-atmospheric pressures and surfactant concentrations. selection of the four most common surfactants: SDS (CMC = 2300 ppm), Tween 20 (CMC = 600 ppm), TritonX-114 (CMC = 120 ppm), CTAB (CMC = 1500 ppm), all of the CMC were measured at room temperature. As shown in Figure 1, after organizing the existing literature using the four-quadrant method, the solid points and semi-solid points, respectively, represent the results of changes in surfactant solution concentration and pressure. It can be observed that the literature lacks research on surfactant concentration under low-pressure conditions. As a matter of fact, under saturated conditions, pressure variations can alter the boiling temperature, subsequently affecting solute solubility and CMC [36]. Additionally, the coupled effects of pressure and surfactants can lead to changes in bubble growth and detachment parameters, such as the gas–liquid density ratio, surface tension, latent heat of vaporization, and the hydrophilic–hydrophobic balance of surfactant molecules. The intensity of bubble disturbances is directly related to heat transfer efficiency. 

According to the above survey, the heat transfer performance after adding surfactants under low-pressure conditions is of great significance for applications in the field of immersion phase-change cooling.

### 1.3. Purpose of the Present Work

As mentioned in Section 1.1, pool boiling enhancement at sub-atmospheric pressure is the primary target in two-phase immersion cooling systems. The role of surfactants in improving boiling performance under low pressure remains unclear. Given the electrical conductivity concerns in practical applications, this study examines the boiling behavior of a non-ionic surfactant (Tween 20) solution under low pressure (8.8–38.5 kPa). Temperature fluctuations from intermittent boiling are analyzed in relation to heat flux, concentration, and pressure. Bubble dynamics—departure diameter, growth time, and waiting time—are evaluated through visualization experiments, and the impact of channel structure on boiling improvement is also discussed.

## 2. Experimental Facilities

### 2.1. Experimental Set-Up

The saturation pressure and temperature of the system must be maintained stable. In this study, a stainless-steel liquid storage tank with a volume of 1 L is used as the container for the working fluid. The outer wall of the storage tank is wrapped with heating bands to control the saturation temperature of the fluid inside the tank, thereby controlling the saturation pressure. The temperature of the fluid inside the tank is measured using T-type thermocouples attached to the wall surface. The pressure inside the tank is measured using a pressure sensor (range 0~2000 kPa). The vacuum pump is connected to the liquid storage tank and the boiling pool via a two-way valve to complete preparations such as vapor removal and fluid flushing. During the experiment, the boiling tank is kept connected to the storage tank, the heating surface is continuously heated by inserting heating rods and generating a phase change, while the condensation coil condenses the rising vapor back into the boiling tank to control the stability of the system’s saturation pressure.

**Figure 2:**
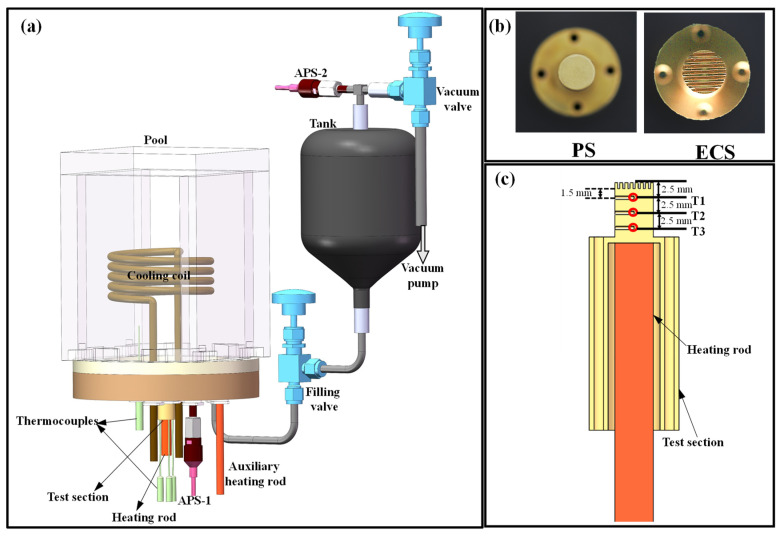
(**a**) The schematic map of experimental setup, (**b**) the image of the PS and ECS, and (**c**) the schematic map of the position of the thermocouples.

As shown in Figure 2a, the boiling pool is constructed from acrylic material in a square space. It includes a condensation coil to maintain stable pressure, a copper block surface for testing heat transfer effectiveness, a hollow cylindrical base for inserting heating rods, an auxiliary heater for maintaining temperature stability, and APS and armored T-type thermocouples for real-time monitoring of pressure and temperature.

### 2.2. Surface Treatment

Before each experiment, detailed surface treatment is performed, including the following steps: (1) alcohol pre-degreasing treatment was carried out, followed by thorough cleaning with DI water to ensure surface cleanliness, (2) copper cleaner (MG440) was used to remove free iron, metallic residues, oxides, and other corrosion products from the surface of the metal, followed by another DI water rinse to ensure surface cleanliness, (3) copper sealer (CS100) was applied to protect the surface, and finally, drying in a vacuum oven to avoid contamination.

### 2.3. Imaging and Measurement

Images of the bubble dynamics behaviors on all test surfaces were captured by a high-speed camera (Phantom LAB110, Wayne, NJ, USA) paired with an autofocus lens (Nikon AF NiKKOR 24-85 mm 1:2.8-4 D, Tokyo, Japan) and a zoom lens (LEICA Z16 APO, Wetzlar, Germany). The main parameters of the high-speed camera were set as follows: frame rate of 1600 fps, resolution of 1280 × 800. Each video was captured for 10 s, totaling 16,000 frames. To obtain sufficiently bright and clear images, a flicker-free LED cold light source (VISICO LED-150T, Yaoyu, China) was used to illuminate the test surface. The image data captured by the high-speed camera were transmitted via Ethernet and saved to a computer. Bubble observation and measurement were conducted using high-speed camera software (Phantom Camera Control, 3.1.772.0, Wayne, NJ, USA).

### 2.4. Heated Surfaces and Solution Preparation

As shown in Figure 2b, both heating surfaces have a diameter of 6 mm and a surface roughness of less than 3.2 μm. In addition, considering the net additional pressure introduced by the microchannels and the flow resistance of the gas–liquid two-phase microchannels, the height of the microchannels was set to 1 mm. The ECS features seven grooves cut into the surface, with a groove width of 0.3 mm at the center and 0.5 mm at the tail. The groove width uniformly widens from the center to the tail. During the experiment, the sides of the copper block were wrapped with 10 mm thick insulation to minimize heat loss to the surroundings, approximating one-dimensional upward heat transfer. As shown in Figure 2c, the heating rod was inserted from the bottom, and three thermocouples were inserted from the top to measure the temperature, evenly spaced in the vertical direction with 2.5 mm intervals and a uniform insertion depth of 3 mm.

The solute is Tween 20 and the solvent is deionized water. A glass pipette was used to draw up 0.1–0.5 mL of solute into 1 L of water, which was stirred for more than 30 min using a magnetic stirring bar to ensure that the solute was uniformly distributed. The experiment should be completed within 4 h after the solution has been prepared to prevent deviations in concentration brought about by the volatilization of water. After completing each set of experiments, the boiling pool and storage tank were cleaned twice with deionized water to prevent solute residue.

## 3. Data Processing and Uncertainty Analysis

To confirm the repeatability of the experiment, two pool boiling experiments were conducted on the same test surface under identical conditions. The HTC curves showed a high degree of conformity, with a maximum difference of only 17%.

**Figure 3:**
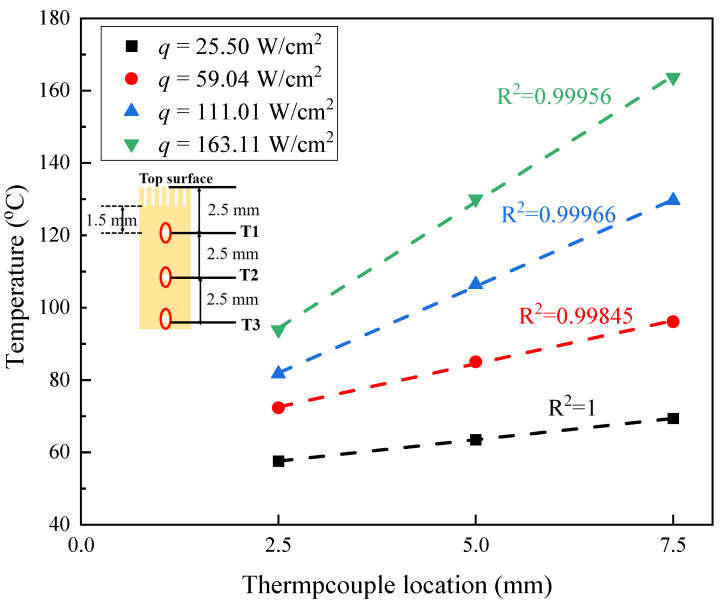
Measured temperature distribution at different heat fluxes.

As shown in Figure 2c, there are three temperature measurement points arranged on the copper block, all positioned along the central axis, labeled as $T_{1}$, $T_{2}$, and $T_{3}$ from top to bottom. Figure 3 shows the temperature distribution during the boiling of deionized water at different heat flux densities. It can be observed that the temperatures of the three thermocouples inside the copper block exhibit nearly linear distribution, with $R^{2}$ values all exceeding 0.99845. 

According to Fourier’s law, the heat flux q is calculated using Equation (1), where $\lambda_{cu}$ is the thermal conductivity of copper. $\frac{dT}{dx}$ represents the slopes of the lines obtained by linear fitting of the temperature points from the three measuring points.

(1) $$q=-\lambda_{cu}\cdot \frac{dT}{dx}$$

The wall temperature $T_{w}$ can be determined using Equation (2), where $z_{w}$ is the distance from the temperature measurement point $T_{1}$ to the heating surface.

(2) $$T_{w}=T_{1}-\frac{q\;z_{w}}{\lambda_{cu}}$$

Finally, the HTC (heat transfer coefficient) is calculated using Equation (3), where $T_{s}$ is the saturation temperature of deionized water under system pressure.

(3) $$HTC=\frac{q}{T_{w}-T_{S}}$$

The sensors and instruments used in this study were calibrated before testing. The main sources of experimental error are temperature measurement errors from the thermocouples and dimensional errors in machining the copper block. The temperature measurement accuracy of the T-type thermocouples is ±0.1 K. The temperature data represent the average values taken over one minute at steady state for each heat flux condition, with a typical input heat flux step size of 10 W. However, to more accurately determine the transition from intermittent boiling to continuous boiling for PS, a step size of 5 W was used at low heat flux in the surfactant solution. The machining accuracy of the distance between adjacent temperature measurement points on the copper block is ±0.1 mm. The accuracy of the pipette is 0.01 of its range. 

After validating the reliability of the experimental setup, for five different concentrations, each concentration was tested three times on two surfaces, eliminating potential interferences such as improper surface treatment and concentration changes due to long-term exposure to surfactants, ensuring the stability and accuracy of the experimental data. Similarly, for five different pressure levels, each pressure was tested twice on two surfaces to ensure that the trend of experimental data with pressure changes remained consistent.

The bubble waiting time was determined through frame-by-frame video analysis, starting from the moment the last bubble leaves the heated surface, and ending when a new bubble appeared after a waiting period. In the 10 s video (16,000 frames), states with a complete waiting process were randomly selected for statistical analysis, and the average result was calculated from these statistics. The statistical minimum bubble wait time was 23 ms and the observed error was 1 frame (0.625 ms). 

The specific calculation methods for determining the maximum uncertainty of heat flux and HTC are provided in Equations (4)–(6). As shown in Table 2, whicn is present maximum uncertainties of measured parameters

(4) $$\frac{U\left(q\right)}{q}=\sqrt{\begin{array}{c} {\left(\frac{\partial (\ln q)}{\partial \left(\lambda_{cu}\right)}U\left(k\lambda_{cu}\right)\right)}^{2}+{\left(\frac{\partial (\ln q)}{\partial \left(\frac{dT}{dx}\right)}U\left(\frac{dT}{dx}\right)\right)}^{2} \end{array}}$$

(5) $$U{(T}_{w})=\sqrt{\begin{array}{c} \begin{array}{c} {\left(\frac{\partial T_{w}}{\partial (z_{w})}U\left(z_{w}\right)\right)}^{2}+{\left(\frac{\partial T_{w}}{\partial \left(T_{1}\right)}U\left(T_{1}\right)\right)}^{2}+{\left(\frac{\partial T_{w}}{\partial \left(\frac{dT}{dx}\right)}U\left(\frac{dT}{dx}\right)\right)}^{2} \end{array} \end{array}\;}$$

(6) $$\frac{U\left(HTC\right)}{HTC}=\sqrt{\begin{array}{c} {\left(\frac{\partial (\ln HTC)}{\partial \left(q\right)}U\left(q\right)\right)}^{2}+{\left(\frac{\partial (\ln HTC)}{\partial {(T}_{w}-T_{s})}U{(T}_{w}-T_{s})\right)}^{2} \end{array}}$$

## 4. Results and Discussion

### 4.1. Effect of Saturated Pressure

**Figure 4:**
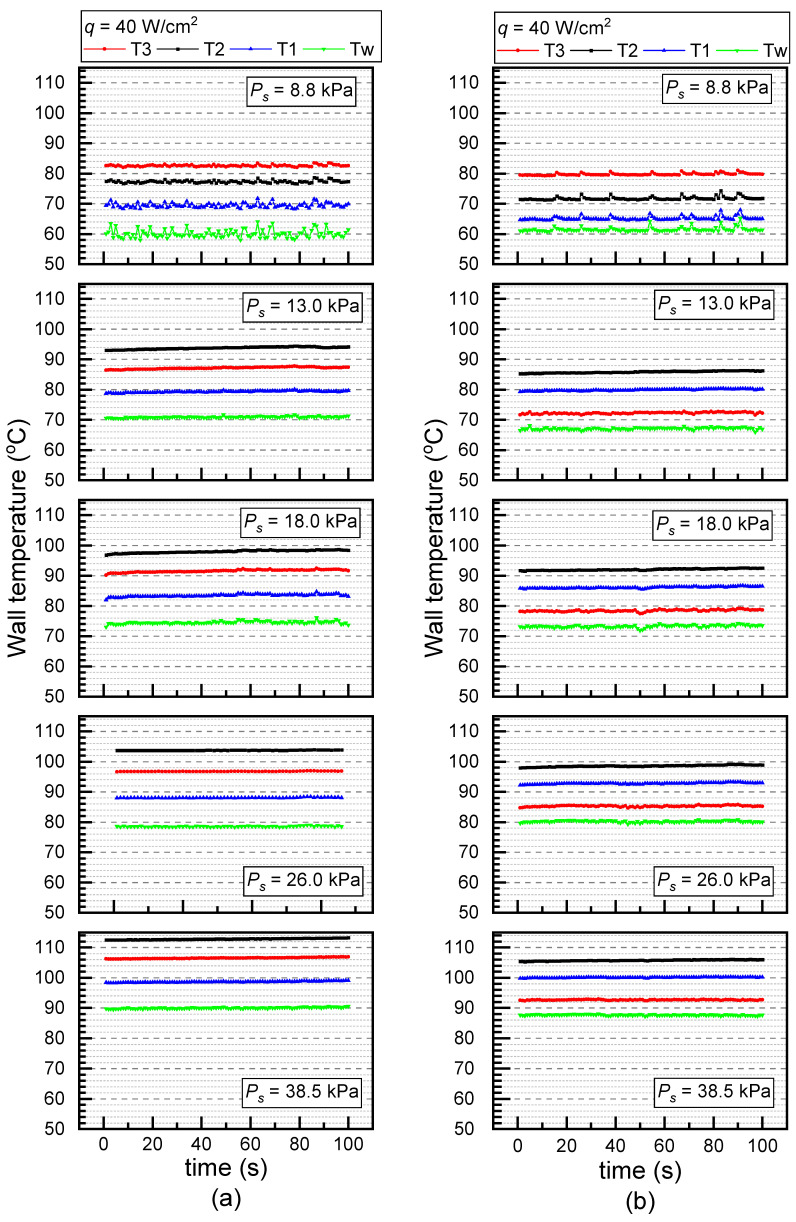
Temperature oscillations at different saturated pressures under *q* = 40 W/cm^2^ and *c* = 0 mL/L (**a**) PS and (**b**) ECS.

Figure 4 compares the temperature oscillations along with the saturated pressure on (a) the PS and (b) the ECS under *q* = 40 W/cm^2^ without surfactant. Increased saturated pressure led to a decreased surface tension coefficient and a reduced Gibbs surface energy. Therefore, the increase in saturated pressure enabled more steady temperature variations on both the PS and ECS. Only slight temperature oscillations were observed at *P_s_* = 8.8 kPa, and barely no oscillation occurred after that on either the PS or ECS. 

**Figure 5:**
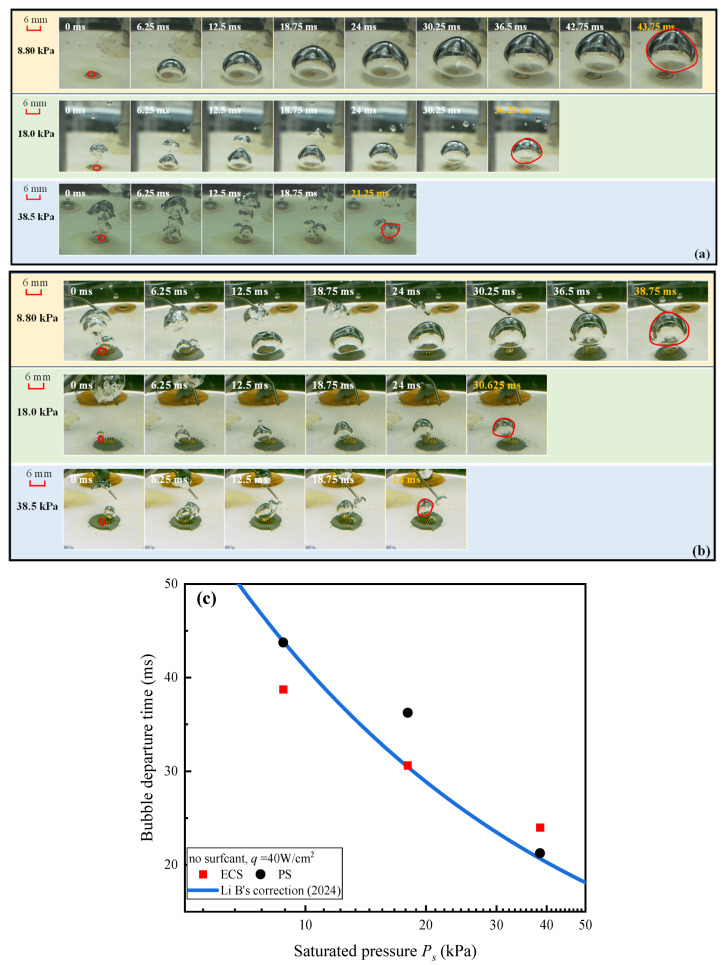
Visual results of bubble growth and detachment on (**a**) PS and (**b**) ECS under *q* = 40 W/cm^2^ and *c* = 0 mL/L, and (**c**) variations of bubble detachment time versus with saturated pressures.

The increased saturated pressure leads to rapidly detached and smaller bubbles. The wall dissipates heat steadily through uniform agitation, resulting in stable temperature variation versus time and no more $t_{w}$ is required. To illustrate this, Figure 5 compares the time for a single bubble to grow and detach on the (a) PS and (b) the ECS under different pressures, and *q* = 40 W/cm^2^. The time step for each frame in the images is 6.25 ms (10 frames), until complete detachment is achieved. According to McGillis’ correlation [17], i.e., $t_{d}=0.266P_{s}^{-0.565}$, where the bubble departure time, $t_{d}$, was in seconds, and the saturated pressure, *P_s_*, was in kPa, the bubble departure time was inversely correlated with the saturated pressure. 

As shown in Figure 5c, as the saturated pressure increased, the time for a bubble to grow from nucleation to detachment gradually decreased. The results basically fit with the tendency change reported in the literature [16,38,55], compared with Li’s correlation [26], for DI water, $i.e.,f{D_{d}}^{0.5}=3.34Ra^{0.15}$, where Ra is the wall roughness, $D_{d}$ is the bubble departure diameter $D_{d}=P^{-1}\sqrt{\frac{\sigma }{\left(\rho_{L}-\rho_{v}\right)g}}$, and since the waiting time of the bubbles is very short, the bubble departure frequency f is considered to be the reciprocal of the bubble departure time. The experimental data fit well with the calculated results. For saturated pressure ranging from 8.8 to 38.5 kPa, the bubble departure time was reduced by 51.4% for PS and 38.0% for the ECS, aligning with the decreasing trends proposed by Li’s correlation.

At the same pressure of 8.8 kPa, the growth time of the bubble on the ECS was less than that on PS, and the detachment diameter was smaller on the ECS compared to PS. However, when the pressure increases to 38.5 kPa, the advantage of the ECS diminishes. This is because the evaporation momentum force as an additional force applied on the bubbles along the detachment direction was inversely proportional to the vapor density. As the saturated pressure decreases, the vapor density decreases, allowing the evaporation momentum force to play a more significant role.

### 4.2. Effect of Surfactant Concentration

**Figure 6:**
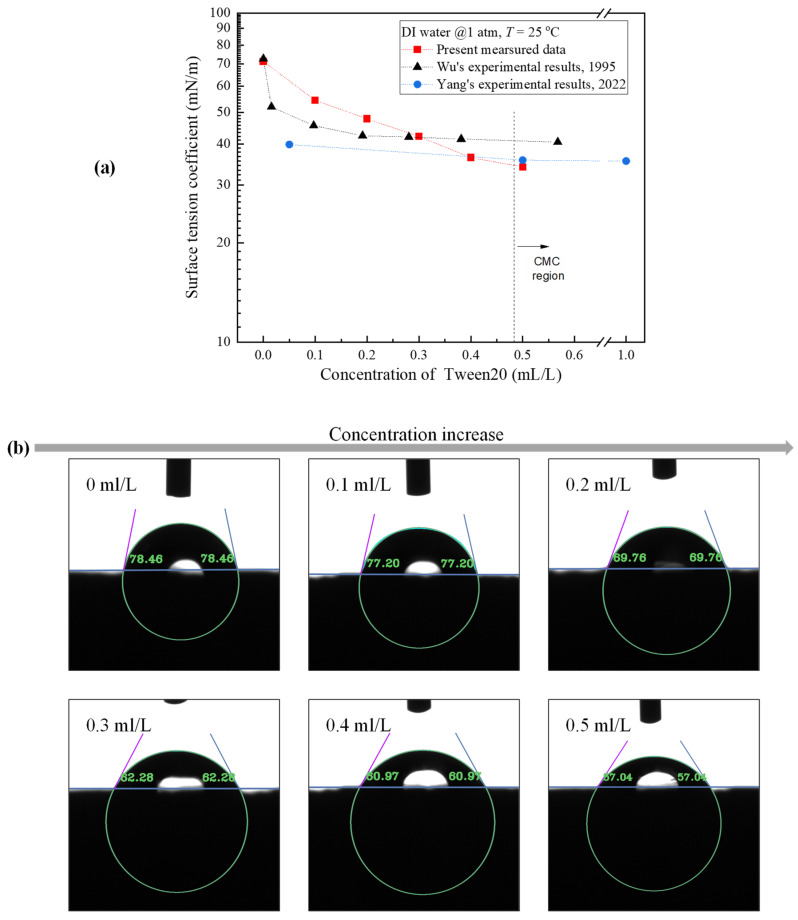
Variations of (**a**) surface tension coefficient and (**b**) static contact angle with Tween 20 concentrations.

Figure 6 displays the variations of (a) the surface tension coefficient and (b) the static contact angle versus the surfactant concentration. The surface tension coefficient was measured by the Wilhelmy plate method at atmospheric pressure. Compared to other researchers [31], the surface tension coefficient generally decreases with increasing concentration. By analyzing the curves of the adjacent data points, it is found that the trend of decreasing surface tension coefficient with increasing concentration becomes gradually smoother, fitted with the reported critical micelle concentration (CMC) of Tween 20 that ranges from 0.48 to 0.6 mL/L [56]. Moreover, the measured static contact angle by using a static contact angle measuring instrument (DSA-X ROLL, Guangzhou, China) varied from 78.46° at *c* = 0 mL/L to 57.04° at *c* = 0.5 mL/L, reflecting the improvement in the hydrophilicity.

**Figure 7:**
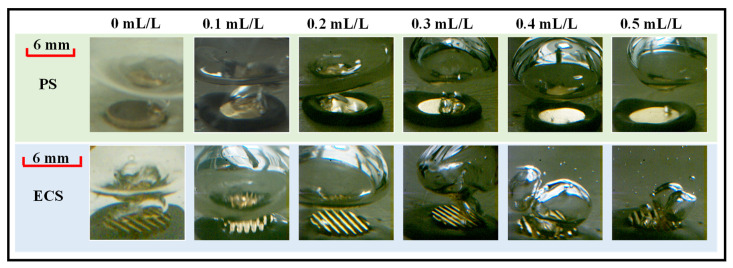
The effect of concentrations on detachment diameters of bubble (*P_s_* = 8.8 kPa, *q* = 40 W/cm^2^).

Figure 7 compares the detach diameters of the bubble on the two surfaces. Despite limitations imposed by the camera’s range of observation, a trend can still be observed: as the concentration increased, the surface tension coefficient decreased, and bubbles were prone to detach, leading to a reduced bubble detachment diameter. The decline trend was even more pronounced on the ECS, especially when comparing the case of *c* = 0 mL/L to *c* = 0.5 mL/L.

**Figure 8:**
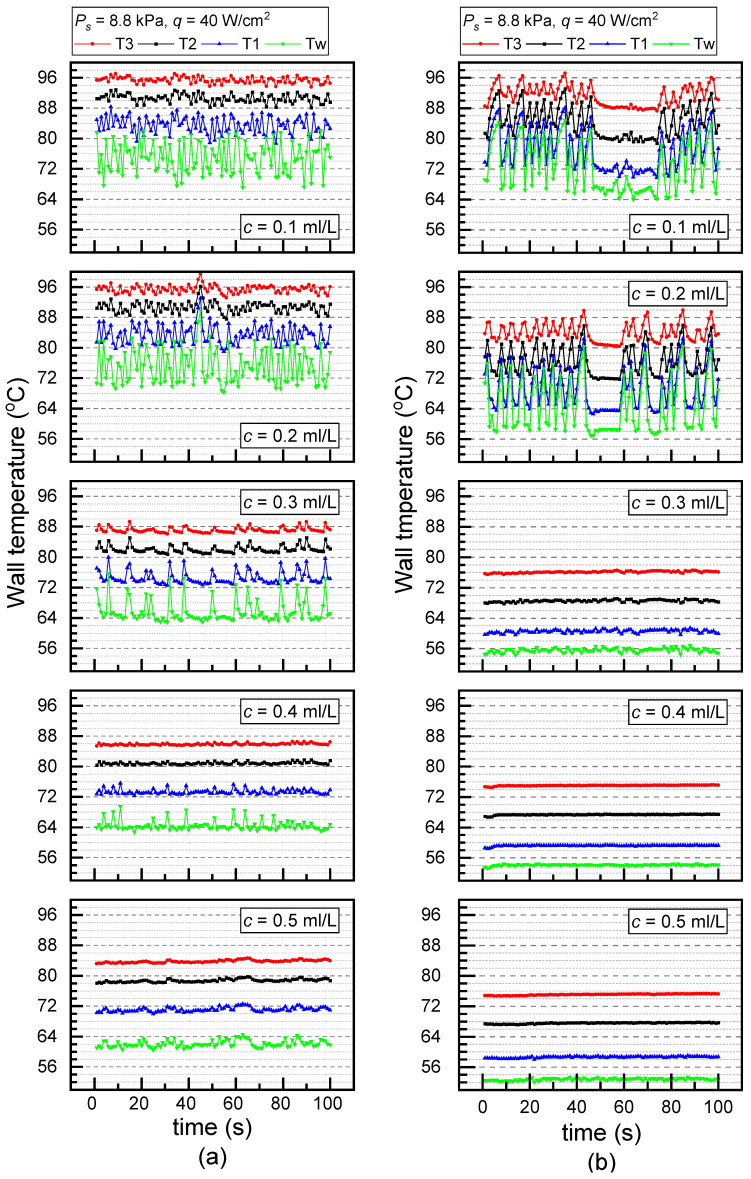
Temperature oscillations at different concentrations under *P_s_* = 8.8 kPa and *q* = 40 W/cm^2^ (**a**) PS and (**b**) ECS.

Figure 8 compared the temperature oscillations along with the surfactant concentration on the PS (a) and the ECS (b) under *P_s_* = 8.8 kPa and *q* = 40 W/cm^2^. At a concentration of 0.1 mL/L, the temperature fluctuations of the PS and ECS reached 10 K and 18 K, respectively. However, when the concentration increased to 0.5 mL/L, the temperature fluctuation of the PS decreased to 2 K, while that of the ECS was less than 1 K. It is shown that the temperature oscillation on either the PS or ECS was significantly weakened along with the increase in surfactant concentration. This is because the Gibbs surface energy for nucleation and bubbling decreased with the increase in surfactant concentration. The temperature oscillations on the ECS and PS were almost eliminated at *c* = 0.3 mL/L and *c* = 0.4 mL/L, respectively.

**Figure 9:**
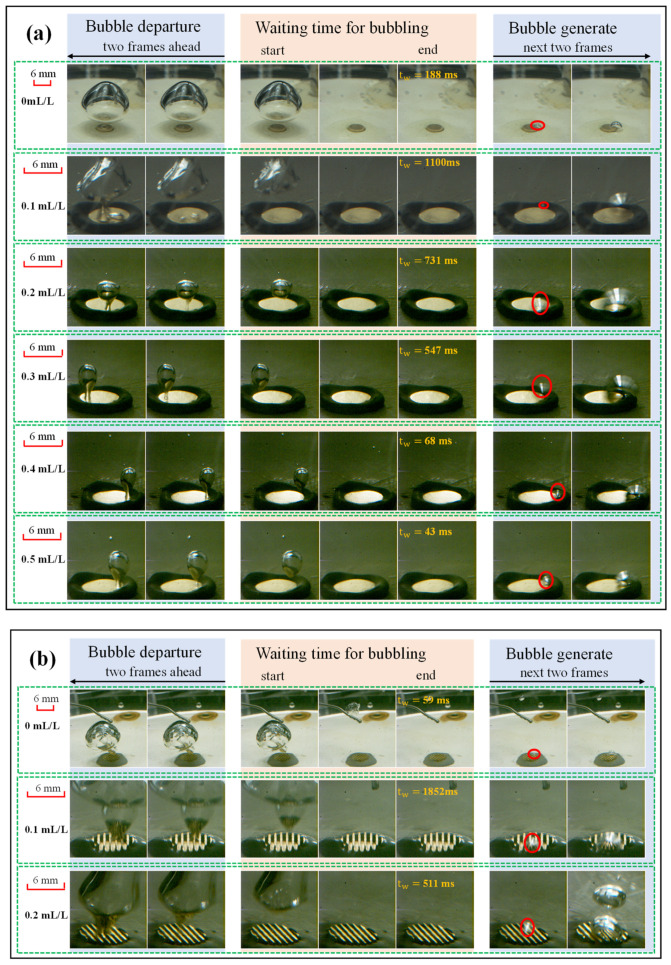
Waiting time for bubbling at different concentration under *P_s_* = 8.8 kPa and *q* = 40 W/cm^2^ (**a**) PS and (**b**) ECS.

The waiting time for bubbling ($t_{w}$) was extended under low saturated pressure so as to accumulate sufficient superheat to overcome the barrier to nucleation. Hence, the $t_{w}$ under various heat fluxes was captured as evidence to illustrate the variation of the temperature oscillation. The waiting time is defined as the time interval from the moment the last bubble leaves the surface to the end of the frame before the appearance of the next bubble. During this time interval, the heat transfer on the surface is only completed by natural convection. Figure 9 compares the $t_{w}$ at different concentrations of (a) the PS and (b) the ECS under *P_s_* = 8.8 kPa and *q* = 40 W/cm^2^. As the concentration changed from 0.1 to 0.5 mL/L, the time intervals were gradually shortened. The $t_{w}$ approached 0 s on the ECS at *c* = 0.3 to 0.5 mL/L, which made it difficult to determine the image of the waiting process in the recorded video as the bubbling and departure process persisted. Comparing the two surfaces at 0.1 mL/L, the $t_{w}$ of the PS is smaller than that of the ECS, but as the concentration increases, the $t_{w}$ of the ECS decreases more significantly. At 0.5 mL/L, there is a constant bubble production on the ECS, while the $t_{w}$ on the PS was 43 ms. Currently, the ECS has the advantage of more nucleation points due to the enlarged area for heat diffusion, which effectively shortens the $t_{w}$.

Comparing the case of adding a surfactant to the one with no surfactants, the results are completely different from the above situations. The added Tween 20 not only changes the surface tension coefficient but also affects the static contact angles. Thereby, the surface became more hydrophilic and unfavorable for nucleation after adding the surfactant, leading to greater temperature oscillations at *c* = 0.1 mL/L than that at *c* = 0 mL/L. Correspondingly, the $t_{w}$ extended at *c* = 0.1 mL/L.

### 4.3. Effect of Heat Flux

**Figure 10:**
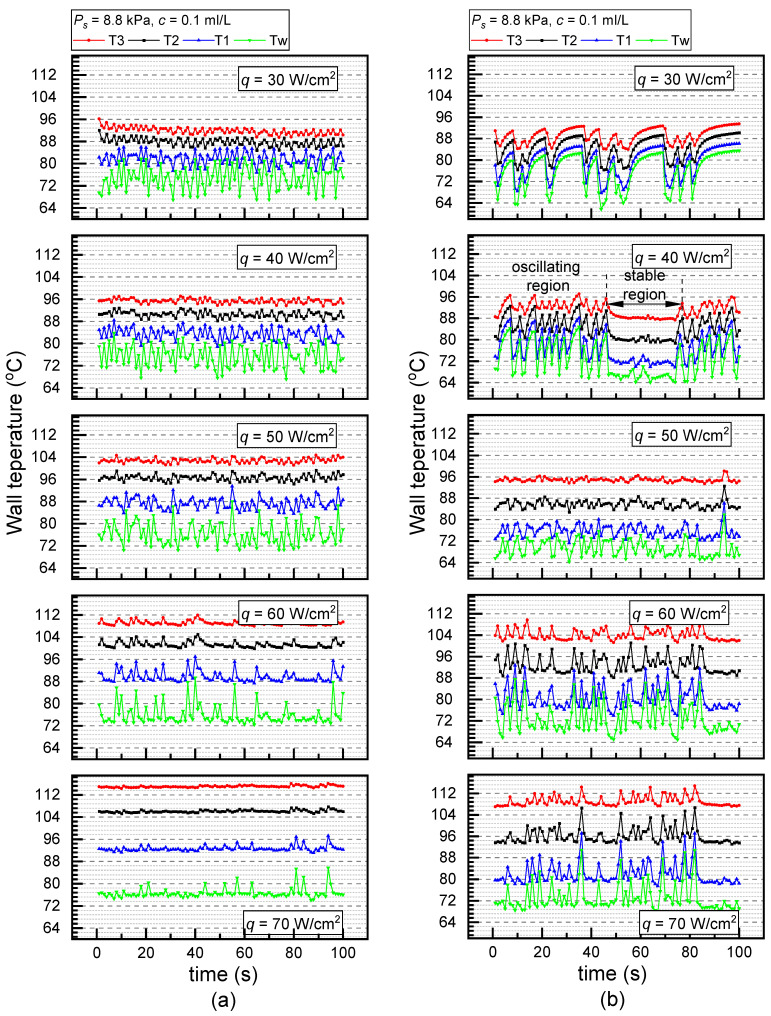
Temperature oscillations at different heat fluxes under *P_s_* = 8.8 kPa and *c* = 0.1 mL/L (**a**) PS and (**b**) ECS.

Heat flux provides extra energy for the nucleation and growth of bubbles. The increase in superheat leads to a higher nucleation rate of bubbles. Figure 10 compares the temperature oscillations along with the heat flux on the PS (a) and the ECS (b) under *P_s_* = 8.8 kPa and *c* = 0.1 mL/L. As illustrated in Figure 10, for both the PS and ECS, the temperature oscillation becomes weakened with the increase in heat flux, illustrating that the intermittent bubbling phenomenon is more pronounced under low heat flux conditions. It is found that the influence of heat flux on temperature oscillation on the PS is more significant than the ECS. For instance, the oscillation amplitude of *T_w_* was about 10 K at *q* = 30 W/cm^2^ and 2 K at *q* = 70 W/cm^2^ on the PS, whereas the oscillation amplitude of *T_w_* was about 14 K at *q* = 30 W/cm^2^ and10 K at *q* = 70 W/cm^2^ on the ECS. This may be because the liquid velocity and forced convection yielded on the channel surface are stronger than on the plane surface. Thus, the temperature oscillation on the ECS is less sensitive to the increase in the heat flux.

**Figure 11:**
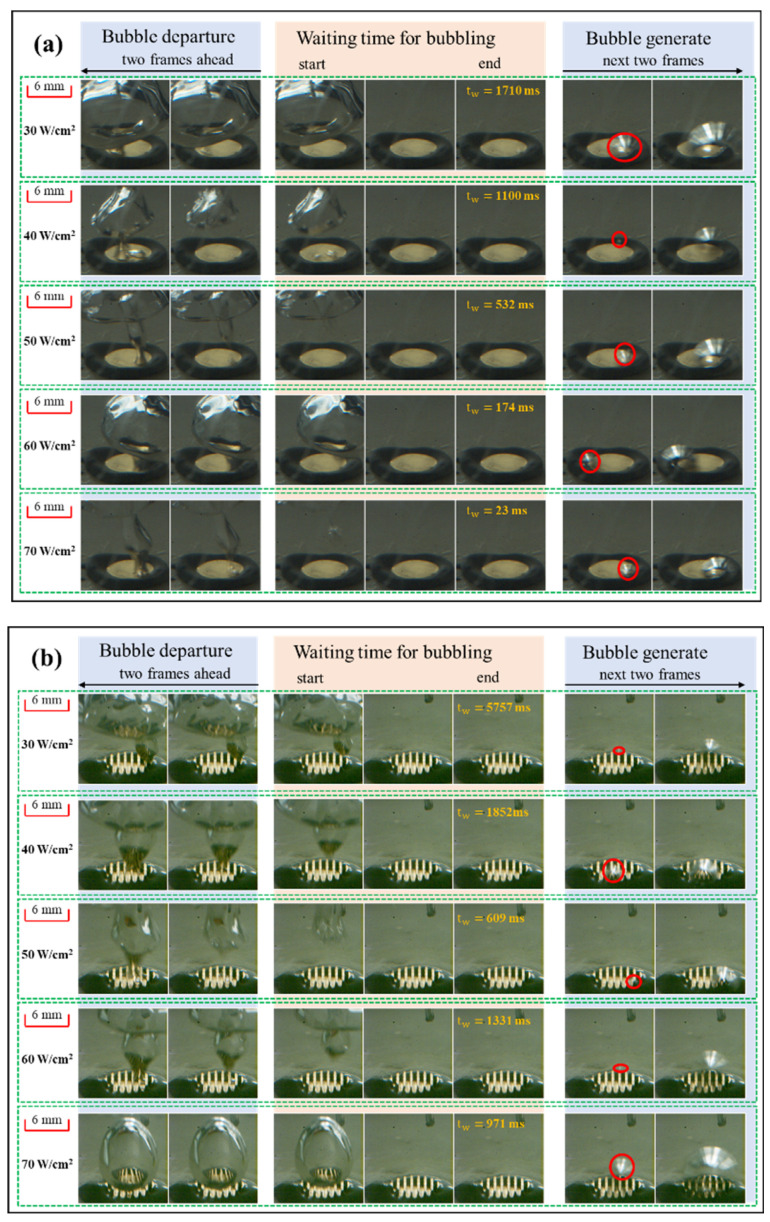
Waiting time for bubbling at different heat fluxes under *P_s_* = 8.8 kPa and *c* = 0.1 mL/L on (**a**) PS and (**b**) ECS.

Figure 11 presents the visualization results of $the\;t_{w}$ under *P_s_* = 8.8 kPa and *c* = 0.1 mL/L. As shown in Figure 11a, the $t_{w}$ on the PS declined from 1710 ms to 23 ms as the heat flux increased from 30 W/cm^2^ to 70 W/cm^2^. The sharp drop in the waiting time corresponds closely with the elimination of temperature oscillation in Figure 10a. Increased heat flux, increased wall superheat, and decreased bubble waiting time are consistent with the negative correlation between waiting time and wall superheat presented by Basu et al. [57]. It should be noted that the waiting time on the ECS at the same heat flux of 30 W/cm^2^ was about 3.4 times longer than that on the PS, as shown in Figure 11b, which is consistent with the aggravated temperature oscillation exhibited in Figure 10b. This aligns with the view that the presence of microchannels leads to enhanced disturbance. Moreover, the waiting time on the ECS was 609 ms at 50 W/cm^2^ and 1331 ms at 60 W/cm^2^, which is accordant with the amplitude enlargement in temperature oscillation under these two cases.

Furthermore, as shown in Figure 10b, at *q* = 40 W/cm^2^, a period of 20 s of stable temperature appeared on the ECS, maintaining the wall temperature of approximately 68 °C at time 50~70 s. Due to smaller bubbles continuously persisting and generated in one channel during the stable period, compared with multiple nucleation cores that were distributed on adjacent channels, they are observed during the temperature oscillation process. This contributed to the decreased length of the triple-phase contact line and the decreased detach diameter of the bubble. In this case, the disturbance of liquid, as well as the temperature oscillation, was relieved.

### 4.4. Boiling Curves and HTC

**Figure 12:**
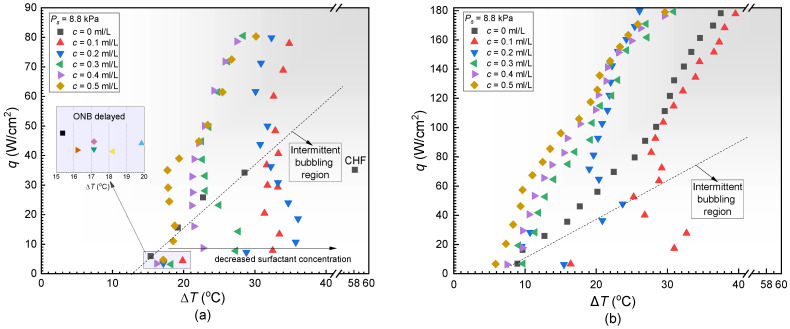
Pool boiling curves under the same saturated pressure (**a**) PS and (**b**) ECS.

**Figure 13:**
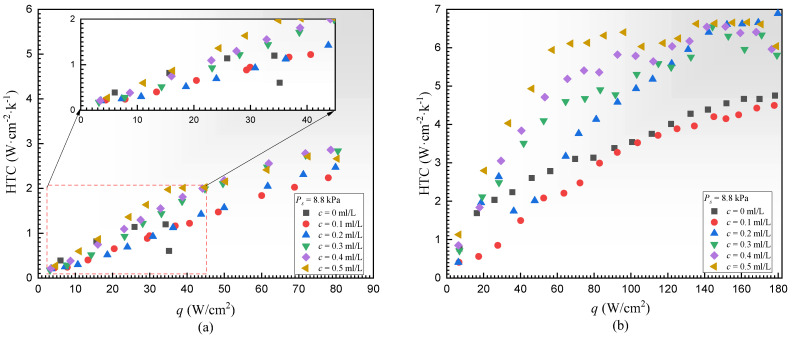
HTC curve under the same saturated pressure (**a**) PS and (**b**) ECS.

Figure 12a plots the boiling curves of DI water with various surfactant concentrations on the PS under *P_S_* = 8.8 kPa, where the black square points refer to the condition without surfactant. It was found that the boiling curve of DI water with surfactant presented as an “S” shape. The superheat Δ*T* increased first and then decreased gradually. The results under sub-atmospheric pressure were different from the atmospheric conditions, as described in [24,31,58]. Compared to the case without surfactant, the heat transfer enhancement brought about by the addition of surfactant only occurred in high heat flux or high concentration regions (>0.3 mL/L). Under low heat flux conditions, severe temperature overshoot occurred when the surfactant concentration varied from 0.1 mL/L to 0.3 mL/L. For instance, at *q* = 10 W/cm^2^, the temperature overshoot at *c* = 0.1 mL/L was about 32.5 °C, which was 17 °C higher than that at *c* = 0 mL/L. This corresponds to the intermittent bubbling behaviors on the heating surface. The temperature overshoot became relieved when the surface concentration increased to 0.4 mL/L. Moreover, at *c* = 0.1 mL/L, the boiling heat transfer improvement did not occur until *q* was larger than 50 W/cm^2^, whereas the boiling enhancement worked only when *q* was larger than 20 W/cm^2^ at *c* = 0.5 mL/L. In addition, considering the dual role of surfactants in reducing static contact angle and surface tension coefficient, the onset of nucleate boiling (ONB) was delayed compared to the case without surfactants, but the order of delay was not exactly consistent with the order of concentration reduction. Moreover, CHF appeared at about 40 W/cm^2^ on the PS when no surfactant was added, but no CHF was recorded after adding Tween 20, indicating the positive role of surfactant in raising the boiling CHF under low saturated pressures. As shown in Figure 13a, the continuous increase in heat flux leads to the maximum HTC reaching 2.86 W·cm^−2^·K^−1^ when *P_S_* = 8.8 kPa, *c* = 0.4 mL/L, and *q* = 80 W/cm^2^, compared to the case where *c* = 0 mL/L. This represents a 138% increment.

Similarly, Figure 12b displays the boiling curves on the ECS under the same conditions. At *c* = 0.1 mL/L, due to the excessively long $t_{w}$, the wall superheat remained consistently higher across the entire range of heat flux compared to the situation without the addition of the concentration, mirroring the behavior observed for the PS. However, as the concentration increases, the heat transfer capability gradually improves, eventually surpassing the case without the addition of the surfactant. For instance, at *q* = 160 W/cm^2^, the wall superheat measured at *c* = 0.5 mL/L was 24.5 °C, which was 10 °C lower than the case when no surfactant was added. As the concentration increased from 0.1 to 0.5 mL/L, the onset of nucleate boiling and wall superheat gradually decreased, and the ONB decreased by 10.54 K. When the heat flux was 160 W/cm^2^ the wall superheat was reduced by 12.8 K. The key difference between the boiling behaviors on the ECS and PS was that the temperature overshoot phenomenon for the ECS only occurred at low concentrations of 0.1 and 0.2 mL/L. Additionally, as shown in Figure 13b, the maximum HTC can reach 6.89 W·cm^−2^·K^−1^, which was increased by 45% compared to the case without surfactant Tween 20.

In summary, the change in heat transfer performance primarily depends on the intensity of the bubble disturbance per unit of time. Under low saturated pressure, compared with pure water, the declined surface tension coefficient still leads to smaller bubbles and increased departure frequency, but it also prolongs the $t_{w}$, causing noticeable temperature overshoot at low heat flux. It should be noted that although there is temperature overshoot at low heat flux when adding surfactant, no CHF is ever measured on the PS or ECS with surfactant added. As the occurrence of CHF requires a persistent vapor-film coverage, the temperature oscillation induced by the “bubble–liquid–bubble” cycle possibly strengthens the disturbance and delays the occurrence of CHF.

### 4.5. Mechanism of Intermittent Bubbling and Temperature Oscillation

Intermittent bubbling and temperature oscillation are observed under low saturated pressure conditions, and the phenomenon gets severe when surfactant is added, which is barely reported in the previous literature. The intermittent boiling and temperature oscillation occurs for two reasons: (1) the increased activation energy of nucleation $∆G_{c}$; and (2) the intensified liquid disturbance during bubble departure.

**Figure 14:**
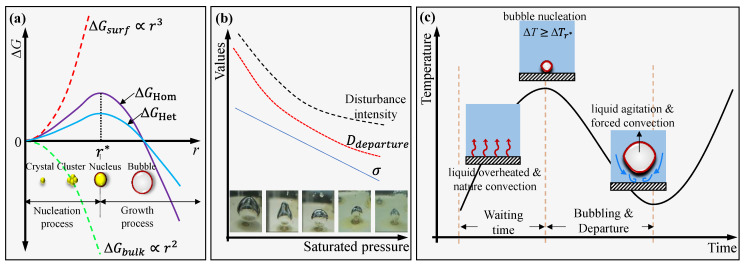
The mechanism of intermittent bubbling and temperature oscillation: (**a**) classical nucleation theory, (**b**) effect of saturated pressure on bubble behaviors, and (**c**) stages of temperature oscillation.

According to classic nucleation theory [59], as shown in Figure 14a, the resistance to the formation of critical nuclei in superheated liquid (surface free energy ${∆G}_{surf}$) is compensated by 2/3 of the decrease in the volume free energy ${∆G}_{bulk}$, while the remaining 1/3 of the resistance is compensated by the energy fluctuation in the superheated liquid. As long as the structural fluctuation of the superheated liquid is ≥$r^{*}$, and the energy fluctuation is $\geq {∆G}_{c}$, the interface between the crystal nucleus and the liquid phase can be formed, and the stable crystal nucleus continues to grow and form a bubble. For heterogeneous nucleation, the required activation energy of nucleation ${∆G}_{c,Het}$ is closely related to the characteristics of the nucleation surface [59], i.e.,

(7) $${∆G}_{c,Het}=\frac{16{\pi \sigma }^{3}T_{s}^{2}}{3(h_{lv}∆{T)}^{2}}f\left(\theta \right)=\frac{16{\pi \sigma }^{3}T_{s}^{2}}{3(h_{lv}∆{T)}^{2}}\cdot \frac{2+cos\theta -{cos}^{2}\theta }{4}$$

where σ is the surface tension coefficient of the liquid, $T_{s}$ is the saturated temperature, ∆T is the superheating degree of liquid, $h_{lv}$ is the latent heat of liquid, and θ is the static contact angle. According to Equation (7), in the case of the same superheating ∆*T*, the decrease in *P_s_* yields an increase in ${∆G}_{c,\;Het}$. Namely, under sub-atmospheric pressure, a larger ∆T is always required for liquid to form nucleation and bubble. As for the surfactant, the adding of surfactant reduces the surface tension coefficient σ on one hand, reducing the barrier to nucleation but decreases the static contact angle θ on the other hand, raising the required activation energy of nucleation. These two aspects are trade-offs in nucleation.

Besides the nucleation, *P_s_* and surfactant concentration also have a significant effect on the departure behaviors of bubbles. As the surface tension is the main force that prevents bubbles from detachment [60], the addition of surfactant benefits the bubble departure. However, the bubble departure diameter is greatly increased under low saturated pressure, reaching 10 times that under atmosphere [61,62]. Considering the large detachment diameter of bubbles at low saturated pressure, as shown in Figure 14b, the disturbance of liquid became vigorous to the heating surface and caused a noticeable temperature drop due to the forced convection.

Figure 14c depicts the development stages of temperature oscillation versus time in one period, where the corresponding behaviors of vapor and liquid phases are also given. Before nucleation, the liquid is overheated to accumulate the energy to activate the nucleus. The wall temperature increases fast in this stage. If the superheating degree of liquid reaches the threshold for nucleation, boiling begins on the surface and the wall temperature decreases. During the bubbling and departure stage, liquid agitation and forced convection also contribute to reducing the wall temperature. The wall temperature continues to decrease and reaches the lowest when the boiling is stopped because of the insufficient liquid superheat. The liquid should be overheated again to be boiled; therefore, intermittent bubbling occurs, and temperature oscillation is observed.

## 5. Conclusions

In this paper, the effects of saturation pressure, surfactant concentration, and heat flux on nucleation, bubble growth and departure, temperature oscillations, and pool boiling enhancement are explored using experimental data. Theoretical analyses were also carried out from the point of view of the energy required for nucleation. The mechanism is proved with visualization evidence and temperature oscillation characteristics. Based on the analysis, the following conclusions are drawn:

(1) Increasing the saturated pressure can effectively promote the transition from intermittent boiling to continuous boiling. In this study, the saturated pressure was systematically reduced from 13 kPa to 8.8 kPa. The decrease in pressure resulted in changes to the increased activation energy of nucleation $∆G_{c}$, leading to a reduction in system stability and making intermittent boiling more pronounced.
(2) At low saturated pressures, the boiling curve of deionized water with added surfactant exhibits an “S” shape. The surfactant reduces both surface tension and wettability, resulting in a more pronounced temperature overshoot at low heat flux compared to without surfactant. However, as the concentration increases, the effects of surface tension become dominant, leading to smaller bubbles that effectively alleviate temperature overshoot. Under 8.8 kPa, boiling enhancement was achieved across all heat flux conditions until a concentration of 0.3 mL/L was reached for the ECS.
(3) The increase in heat flux allows for more heat transfer to the surface for nucleation, which can shorten the waiting time for bubbling under sub-atmospheric pressure. The maximum HTC of the PS with added surfactant reached 2.86 W·cm^−2^·K^−1^, representing a 138% increase compared to deionized water. The maximum HTC of the ECS with added surfactant reached 6.89 W·cm^−2^·K^−1^, which was increased by 45%.
(4) When the pressure decreased from 38.5 kPa to 8.8 kPa, the bubble detachment time significantly increased due to a sharp increase in bubble size, extending by 105% for the PS. At this point, with the addition of a low surfactant concentration, more pronounced intermittent boiling and temperature oscillations occurred. When the concentration decreased from 0.5 mL to 0.1 mL, the bubble waiting time increased by more than 25 times.

In summary, the expanding microchanneled surface effectively intensifies the bubble removal capability and enhances pool boiling heat transfer performance, which is suitable for low-pressure two-phase immersion cooling of DI water. A higher surfactant concentration is recommended to mitigate temperature oscillations in sub-atmospheric pressure applications.

## Figures and Tables

**Table 1:** Overview of research on pool boiling under various pressure ranges and with surfactants.

**Reference**	**Pressure Variation (kPa) ^a^**
Luling Yuan (2019) [37]	12.3, 31.2, 101
Sandra Michaie (2017) [38]	4.2, 5.6, 7.4, 9.6, 12.4, 15.8, 20, 25.1, 31.2, 38.6, 47.4, 57.9, 70.2, 84.6, 101.4
Tomasz Halon (2017) [39]	0.75, 1, 2, 4,
Sang M. Kwark (2010) [40]	20, 47, 101, 200
Xiang Wang (2024) [41]	40, 60, 80, 100, 120
Ayşenur Ateş (2023) [42]	28.3,103.7,
Guanghan Huang (2022) [43]	25, 65
Aniruddha Pal (2008) [44]	9.7, 15, 21
Smreeti Dahariya (2019) [45]	103.4, 206.8, 310.2, 413.7
Hanzhi Chen (2017) [46]	150, 200, 300, 400
Staniszewski (1959) [47]	101, 193, 276,
Akiyama (1969) [48]	101, 203, 507, 807
**Reference**	**Concentration Range (Ratio to CMC) ^b^**
Bing Li (2024) [26]	0.1, 1, 5, 10 (SDS)
Sasan Etedali (2019) [49]	0.480 (SDS) 0.732 (CTAB)
Chi Young Lee (2014) [29]	0.437 (SDS)
R.I. Elghanam (2011) [18]	0.087, 0.217, 0.435, 0.652 (SDS)
Zi-Cheng Hu (2022) [50]	0.087, 0.174, 0.348, 0.435, 0.783, 1.3 (SDS)
Jialun Yin (2020) [51]	0.135, 0.405, 0.946 (SDS)
Zhen Yang (2022) [31]	0.093, 0.93, 1.85 (Tween 20)
Mario R. Mata (2022) [52]	(0.02~31.3) (Tween 20)
H. Jeremy Cho (2013) [53]	0.61 (TritonX-114)
Dong-Sheng Guo (2020) [54]	1 (TritonX-114)
Tao Wen (2022) [25]	0.267, 0.533, 1.066, 2.133 (CTAB)

^a^: the works in the column are all for pure liquid (Key-116 was used in [41], methane was used in [46], others: DI water). ^b^: the works in the column are all at 101 kPa.

**Table 2:** Maximum uncertainties of measured parameters.

Parameters	Maximum Relative Uncertainty
Heat flux (W/cm^2^)	16.9%
HTC (W/cm^2^·K^−1^)	20.6%
Concentration of surfactant	1.0%
Static contact angle (^o^)	1.4%
Waiting time of bubble (ms)	2.7%

## Data Availability

The original contributions presented in the study are included in the article, further inquiries can be directed to the corresponding author.

## Nomenclature

*C*	surfactant concentration, mL/L
*G*	energy
$h_{lv}$	latent heat of liquid, kJ/kg
*P_s_*	saturated pressure, kPa
q	heat flux, W/cm^2^
*r*	radius, mm
*T*	temperature, K
Δ*T*	superheat, K
$t_{d}$	bubble departure time, ms
$t_{w}$	waiting time for bubbling, ms
Ra	wall roughness, um
**Greek alphabet**	
θ	static contact angle, °
σ	surface tension coefficient, mN/m
**Abbreviations**	
CTAB	cetyltrimethylammonium bromide
ECS	expanding microchanneled surface
ONB	onset of nucleate boiling
ppm	part per million
PS	plane surface
SDS	solution sodium dodecyl sulfate
Tween 20	polysorbate 20
TritonX-114	Tert-OCLylphenoxy poly ethanol
